# Older workers and extended working life – Managers’ experiences and age management

**DOI:** 10.3233/WOR-230468

**Published:** 2024-11-08

**Authors:** Anita Björklund Carlstedt, Cecilia Bjursell, Rosita Nyman, Anna Dahl Aslan

**Affiliations:** a Department of Rehabilitation, School of Health and Welfare, Jönköping University, Ageing Research Network – Jönköping (ARN-J), Sweden; b School of Education and Communication, Jönköping University, Sweden; c School of Health and Welfare, Institute of Gerontology, Jönköping University, Ageing Research Network – Jönköping (ARN-J), Sweden; d Institution of Health Sciences, University of Skövde, Sweden

**Keywords:** Competence transfer, competence development, knowledge gaps, prolonged working life, transition

## Abstract

**BACKGROUND:**

In 2020 the Swedish Government started a gradual raising of the retirement age, but employers have been silent on the issue. Little is known about whether and how they reflect on what it will mean for their organization, or whether they already have, or are going to, make arrangements in order to facilitate and motivate older workers to stay longer.

**OBJECTIVE:**

The aim of this study was to explore and describe managers’ experiences of older workers and age management in connection with the increase of the retirement age in Sweden.

**METHODS:**

Data was collected through semi-structured interviews with fourteen managers from a broad set of organizations in the public and private sectors, and from the Middle and East of Sweden. The transcribed material was analysed in line with qualitative content analysis.

**RESULTS:**

The analysis ended up in seven main categories with associated sub-categories: *Older Workers, Retirement Ages, Transition Initiatives, Competence Transfer, Competence Development, Increased Retirement Ages, Knowledge Gaps.*

**CONCLUSION:**

Our findings reveal that there is an ambivalence in addressing the issue of age among the interviewed managers, what we have interpreted and labelled as “silent age discrimination”, and it was shown that they do not have elaborated strategies for age management.

## 1 Introduction

Skills shortages, coupled with demographic changes that are leading to a larger proportion of older adults in the population, have raised the issue of extending working lives in several countries [1]. This is addressed in global and national policies, but as Wainwright et al. [2] found there could be gaps between policies and practices in organizations. Alongside the good intentions contained in governmental policies, there seems to be moderate interest within organisations to make special arrangements for older workers in other national contexts as well. This calls for knowledge and practices that can guide employers in retaining qualified staff and enable and motivate them to perform throughout a prolonged working life.

Age management is suggested as one way to conceptualize organizational practices related to age in various ways. Broadly, the concept refers to the management and retainment of employees who are approaching retirement age, although it has been shown to benefit all age groups [3]. The ambition of age management is to consider the different strengths of a diverse work force [4]. Age-friendliness in workplaces is another way to conceptualize policies and practices that support ageing workers [5]. Flexible working arrangements (FWAs) are a model for retaining older workers, but the value of this as a retention tool does not vary significantly with age [6].

It is furthermore well documented that managers’ attitudes play an important role in whether, and how, age management practices are implemented. Managers’ attitudes to and perceptions of older workers range from the positive [2, 7–9] to negative attitudes and perceptions towards older workers, seeing them as inflexible, IT incompetent and having difficulty learning new things [2]. It is likely that positive and negative attitudes coexist in organizations [10] and the age of a manager influences the attitude towards older and younger workers, as managers tend to judge workers of a similar age to themselves more positively [11, 12]. Managers who have experience of managing older workers are more likely to be open to involvement. Considering the influence that managers have on employee retirement decisions, this calls for training to help managers understand their own role in supporting older employees’ careers [12].

A hindering factor for an extended working life is age discrimination, both the legal dimension concerning behaviours that are prohibited by law, and the social dimension which refers to those actions that occur in the interpersonal sphere [13, 14]. There are a number of practices to deal with age discrimination in working life, such as recruitment processes, training and mentoring opportunities, education for managers and employees in the workplace, flexible employment opportunities, job autonomy, recognition and respect, supportive organizational climate and social support [15, 16]. Still, managers and older workers often ignore the issue of age and ageing in the workplace, and maybe because of this, age has not yet been addressed effectively in working life [1].

Interestingly, HR practices have been found to be weak predictors of older people participating in working life [17]. This could stem from older workers perceiving age-aware HR practices as negative, since they may be associated with negative stereotypes associated with older workers and signal that they belong to a stigmatized group in society. Supportive attitudes from employers and colleagues could play a more prominent role in changing older workers’ decisions to stay in working life.

In 2020 the Swedish Government started a gradual raising of the retirement age [18], from 65 to 66, and from 67 to 68. Since there are both social and economic incitements for a longer work life, together with improved health measured in decreased mortality and self-reported health, more people could work longer if the right prerequisites are in place. From a previous review [] we know that individuals working after pensionable age are often in good health and interested in making money, and that there are positive associations between education level and participation in both work and education later in life [20]. These studies show that it is possible to extend working life and in the national context at hand, there is an ongoing increase of retirement age which could make the topic more urgent. Managers’ attitudes and norms are central, since they are doorkeepers to the human and financial resources that contribute to the development and retention of older employees [21–23].

## 2 Aim

The aim of this study was to explore and describe managers’ experiences of older workers and age management in connection with the increase of the retirement age in Sweden.

## 3 Material and method

The design of this study is descriptive. Semi-structured interviews [24] were conducted with fourteen managers and the data was analysed with qualitative content analysis [25].

### 3.1 Informants

Fourteen managers from the Middle and the East of Sweden were recruited by purposive sampling to reach a variation of their experiences from different branches, and both private and public companies, see Table 1. The recruitment process was based on personal acquaintance of the authors who asked for presumptive informants in the current companies.

**Table 1:** Interviewed persons 1–14 versus interviews 1–13

Interview	Person	Job title	Branches	Organization	Age
1	1	HR manager	Construction industry	Private	40
2	2, 3	HR managers	Social administration	Public	45, 58
3	4	HR manager	Recruitment industry	Private	43
4	5	Manager	Municipal administration	Public	64
5	6	Manager	Police authority	Public	45
6	7	Manager	Training company	Private	40
7	8	Manager	Computer company	Private	55
8	9	HR manager	Construction industry	Private	52
9	10	HR manager	Manufactoring industry	Private	52
10	11	Recruitment manager	Recruitment industry	Private	25
11	12	HR manager	Manufactoring industry	Private	50
12	13	HR manager	Health care company	Public	55
13	14	Manager	County Council	Public	60
m = 48; sd = 9

### 3.2 Interview guide

An interview guide was constructed around five thematic areas, see Appendix 1, to address practical issues in the organization connected to retirement in various ways, as this was a current topic in society at the time of the study.

### 3.3 Data gathering

The interviews were performed in late 2019 and the first months of 2020, before the outbreak of the Covid pandemic. The interviews were performed at the informants’ workplaces. Each interview lasted 60–75 minutes. The interviews were recorded and transcribed verbatim.

### 3.4 Data analysis

The transcripts were read through several times to gain an overview of the content. The text was structured in relation to the five question areas from the interview guide and analysed in line with manifest qualitative content analysis in line with Graneheim and Lundman [25]. Meaning units in relation to each question area were marked, condensed, coded and categorized. The five question areas generated seven main categories, with associated subcategories, and were transformed to descriptive text illustrated with quotations from the interviewees. (A table of main categories and subcategories is available through the corresponding author.)

### 3.5 Ethical considerations

This study was performed in line with the Swedish Research Council’s [26] recommendations for good research practice and the European Code of Conduct for Research Integrity [27]. The rules for protection of personal data were also followed. The informants were informed about the aim of study, the study context, and their right to withdraw participation at any time without explanation. The use of data and confidential storage at the current university was standardized according to legal demands and information about this was given to the respondents. Written informed consent was obtained.

### 3.6 Trustworthiness

To strengthen the trustworthiness of our study we considered the four aspects from Lincoln and Guba [28]. *Credibility* of the study was strengthened by the varied selection of informants, the deep and extensive data material, and a detailed description of the analysis process. Since the data analysis was conducted by the first author alone, consequently the other authors took part of the recorded interviews to secure *confirmability*. Additionally, the findings were backed up by selected quotations to verify the analysis. *Dependability* concerns how stable the results are over time, and a limitation was that the interviews took place before the outbreak of Covid-19 in 2020. To strengthen the dependability of the findings, it was thoroughly discussed among the authors and in relation to other studies and theories. The description of the study context and professional backgrounds of the informants may facilitate the *transferability* to similar study contexts, at least in Sweden.

## 4 Findings

The structure of the findings follows the seven main categories with subcategories: *Older Workers in the Organization, Retirement Ages in the Organization, Transition Initiatives, Competence Transfer, Competence Development, Increased Retirement Age, Knowledge Gaps. *Selected quotes illustrate the informants’ comments. It should be noted that the numbers within parenthesis stand for interviews 1–13, not the informants (14).

### 4.1 Older workers in the organization

In the interviews, the informants reflected on when age is an issue in the organization and what characterizes an older worker. The informants stated that they do not discuss age in recruitment processes, but usually in relation to upcoming retirement.

*No, we do not discuss in those terms at all* ...   *we do not make any distinction at all in our recruitment processes, it rarely comes up, possibly if we start discussing whether someone should retire and how we should replace that person, but it is the only time the age question comes up. (11)*

Describing an older worker was done in relation to age limits, experience, and functional ability. Age limits referred to the common retirement age. When asked about when an employee is considered old, most informants mentioned 60 years and older. They also stressed that they prefer to talk about an older worker as a person with longer experience.

*When we talk age, it*’*s a lot about experience, also that they think of an older person, and then it does not have to be someone old in age but someone who is older (than they are). (10)*

### 4.2 Retirement ages in the organization

When it comes to retirement, differences between professional groups were emphasized by the informants. Workers with high physical demands, so called blue-collar workers, retired earlier while white-collar workers and specialists retired later.

*There are those who go before 65 but there is still quite a lot of focus on 65. Among blue-collar workers, there are probably some who have left earlier, maybe because their body is worn out, but otherwise I think that if you have the opportunity, like on the white-collar side, I think it tends to be later. We probably have someone who have left earlier but we also have someone who should have retired a long time ago. (9)*

There were also examples of workers who stayed longer than the stipulated retirement age:

*We don’t have that many people who have retired but...yeah, I don’t have numbers on that, but there are some people who wants to go earlier, and it has mainly been workers and then there are officials who have chosen to stay until 67 so it has probably been a bit of everything, 63, 64 to 67. (1)*

Differences in relation to occupations were put forward by the informants. An example was teachers who worked beyond retirement age:

*Some work beyond retirement age, after 67, certainly in schools and so on, pensioners are hired and that is managed by HR. (4)*

### 4.3 Transition initiatives

No certain strategies or routines for transition from work to retirement were put forward by the informants, and the transition process was initiated either by the employee, the manager or HR. Most commonly, the HR department dealt with the issue.

*No, there are no routines for that, and when it happens, there is just a conversation,*  ...   *you sit and talk about it. (7)*

*It*’*s the responsible manager* ...   *it should be, but I think HR has been involved quite often, they (employees) have gotten into the habit of going to the HR manager to talk about such things. (9)*

The informants indicated that the year before the official retirement age was the most common occasion when the question of retirement was raised, usually at the yearly employee evaluation. Another approach mentioned by the informants was to send a letter to the employee, but in many cases, this was considered unpleasant or even offensive by employees.

*We have a duty to leave when you turn 67 and then you receive a letter about this, which many people found unpleasant. It feels like I am not worth anything anymore, I must quit. We have realized that it could be presented in a different way. (2)*

Adjustments to age was available in some cases according to the informants. Before retirement, it was possible to reduce the hours of work to 80% and get 90% of the salary and 100% occupational pension. This measure was aimed at enabling a longer work life in conjunction with wellness initiatives. After retirement age it was possible to work according to special arrangements and framework agreements. One of the informants mentioned a system of retirement education, where employees could prepare themselves for retirement.

*I do not remember what age you are invited* ...   *you get a letter home at a specific age, so there is a system to prepare to retire. (12)*

### 4.4 Competence transfer

The informants stated that the organizations often lacked systems and routines for competence transfer. The ambiguity and lack of planning ended up in a loss of competence and experience when older workers retired.

*I think you must deal with it when it comes to a person who is about to retire* – *that you start talking about it early so that the next person does not have to come in and there will be this skill gap. (9)*

To enable competence transfer, certain activities were suggested by the informants, like having planning systems and getting started in good time.

*We are trying to have a plan and the person I mentioned before, who will work here for a couple of more years, we have already started to talk about the customers and the relationships and who you work with in that role, who can accompany you on customer visits. (1)*

Competence transfer seemed to be implemented on a case-by-case basis. Practices used were shadowing someone during ordinary work or mentorship. The reason for implementing competence transfer was the insight that older workers have a great deal of knowledge and skills that were needed in the workplace.

*If I must have a replacement for him, then maybe they should work in parallel with him for the last six months to share knowledge and experience, so there are many such practical questions. (5)*

### 4.5 Competence development

The initiative for competence development of older employees was taken in the yearly employee appraisal, as well as continuously, over the course of a career from junior to senior employee.

*We have worked to find who can take the next step and then we also look at how many people that will probably retire, so we have the supply of skills from the time you are new here to the time you retire, so we do work on it. (8)*

Implementation of competence development focuses on professional programmes, but also on general issues such as healthy work environments and digitalization. Competence development is given both by internal and external performers. Even though the informants stressed that they have both systems and material for competence development, some of them seem rather uninitiated in what it is all about.

*So, we have a folder on the intranet where there are an incredible number of procedures and there is also, if you go in there and check, I am completely convinced that there are skills development parts that you can read about. Cannot say that I am super-savvy. (10)*

Organizational or individual needs, and position govern the individual possibilities for competence development, and a personal plan for development often must be worked out.

*I would probably say that it (digitalization) is a question that is very much one for older workers and where you must do almost an individual survey, where is each employee in their digital process, some are far ahead of the company while some can barely open an email. (6)*

### 4.6 Increased retirement age

When the informants reflected on the governmental increase in the retirement age in 2020, they stated that there were no discussions at the workplace; it was regarded as “what happens happens” or as an HR issue.

*I think that this is an issue for HR. So, they probably did it there but it*’*s nothing I*’*m aware of. I have not received any information. (6)*

There were informants that speculated about whether the rise in the retirement age might change attitudes towards ageing. Sixty years becomes further away from the retirement point, and a sixty-year-old person is no longer considered old. Other advantages they put forward were freedom of choice regardless of age.

*I think it will be a psychological effect, maybe 60 is not so old suddenly*  ...   *I think it can have mental effects that make you value age in a different way so I think it can be positive. So, I think it can be positive and it is positive in the way that I think it should be a great freedom of choice for the individual to do what you want, if you want to work for a long time, then there should be opportunities for it. (3)*

A societal challenge put forward by the informants could be an increase in sick leave because of physically demanding work situations for employees who do not have the physical fitness to go on working. For employers, a challenge lays in handling the situation when workers want to go on working but are not able to fulfil the demands of the work anymore. The informants mentioned it could be a challenge to the individual employee feeling forced to work longer for financial reasons.

*Then we concluded that there is no longer any age when you should stop working* ...   *but you must work until you die* ...   *and we saw this as a difficult problem to be able to terminate an employment in a respectful way. (12)*

The informants, managers and HR managers, were awaiting directives from the authorities, and they had received some employment law information. Altogether, they were aware that sustainable conditions had to be created and structural changes made.

*We need to do our homework to be able to keep people (in employment) until the age of 68 by influencing and improving the work so that it is sustainable in the long run. (9)*

### 4.7 Knowledge gaps

When asked what kind of knowledge they needed or would like to have, the informants mentioned knowledge about work ability assessments, laws, and regulations, how to retain older workers, gerontology, risk analysis, methodology development and mapping of competences.

*We must think about work environment training where everyone gets involved and try to see risks and how we can improve so that everyone is sustainable and strong until higher retirement age. I think we need to get better at it. Then I think we need to become better at mapping skills so that we use skills better. (9)*

The informants emphasized a lack of knowledge about national as well as global laws and regulations concerning termination of employment.

*If you go from Sweden to Denmark, there are completely different employment agreements and employment conditions, the safety net is not at all the same down there. What do we do when we start working across borders then? Which employment agreements do we apply, and which laws and regulations should we follow* ...  *? (10)*

The informants also expressed a need for knowledge about how to make a sustainable working life possible, as well as what shapes good conditions and organizational structure to keep older workers.

*It*’*s so much about sustainability and the conditions offered, what structures are there in the organization to be able to take care of an employee who will stay in the organization for a long time. (3)*

Further, the informants recognized that there were prejudices about older workers, but they were not sure how to address this issue.

*What makes us think that a 35-year-old is much more productive than a 55-year-old or 60-year-old, what do we base that on? Where did we get it from? And to turn it around, we need new research that presents new evidence so that we can break down these prejudices. (3)*

Brain function and signs of ageing were other knowledge gaps that were identified and discussed, together with the current challenges of digitalization and globalization.

,...   *maybe some education about how the brain works when you get older so that, as employers and others, we get a little more insight. (13)*

## 5 Discussion

Against the background that many countries face an ageing population, and that government are looking at changes in retirement age, this study have explored statements from managers and HR managers in Sweden to deepen the knowledge about what is being done in organizations to adapt to these changes.

Firstly, we had an interest in how managers addressed the issue of age in organizations. When asked about who was regarded as an “older worker in the organization”, the informants in this study established that age was rarely discussed in the organizations, and they preferred to talk about work life experience rather than age. This could be interpreted to express oneself in a politically correct way, as we have laws in Sweden prohibiting age discrimination, but it could also be interpreted as if managers ignore the issue of ageing in the workplace. They do, however, in line with Egdell et al. [1] recognize the needs for skills as well as the policy discussion about a raised retirement age. The fact that informants ignore the issue of ageing in the workplace, as our study indicates, can be one explanation for why age has not yet been addressed in policies or practices [1]. The informants in the present study did, however, initiate discussions about *retirement* with employees, but initiatives that concert the *transition to retirement* were usually managed by HR. However, they described the communication as being performed quite mechanically in the year before the official retirement age. They gave examples of practices were people received emails with information about retirement, rather than face-to-face discussions about options for the future. This is in line with van Dalen et al. [8] who established that employers tend to use exit policies and promote early retirement, rather than encourage career development and training for older employees. Still, regarding the discussion about changed demographics and raised retirement ages, this is noteworthy since HR practices were found by several researchers, as Dello Russo et al. [30]; Innocenti, et al. [31]; Karpinska et al. [32]; Kooij et al. [33]; Korff, et al. [34]; and Polat et al. [35], to have a strong influence on older workers in terms of, for example, performance, job satisfaction and motivation.

Secondly, we have tried to identify what is being done to enable an extended working life. The informants in this study identified a lack of routines and systems for *competence transfer* between older and younger workers, even if some had developed practices for mentorship and training. Nevertheless, they recognized the benefits of systems för planning and management of the last part of a person’s career. Connected to this, they did not use *competence development* as a tool for supporting a prolonged working life. Furthermore, they confessed that competence development was unevenly distributed among employees, with younger employees, and employees in management and expert positions receiving the most competence development. This despite the facts that Liu et al. [36] and Naghavi et al. [37] found competence development, in terms of education and training, as an established way to support, develop and retain older workers. There was, however, no mention of models such as “age-friendly workplaces” put forward by Eppler-Hattab et al. [5] or “flexible working arrangements” described by Stirpe et al. [6]. Instead, the current study finds a lack of efforts to develop and retain older workers.

Finally, in addition to notions about age and systems to support an extended working life, we also asked the informants specifically about the current policy changes. In the last two main categories, *Increased retirement age* and *Knowledge gaps*, the answers indicated different attitudes towards age and older workers. The comments went from being oblivious and seeing managing of older workers as an HR issue, to being very positive and seeing advantages in the higher retirement age as it would raise the bar for who was considered old. There was clearly an ambivalence among the informants in how to relate to the issue of age, and as Zientara [2] and Fuertes et al. [10] put forward it is not uncommon to find both negative and positive attitudes coexisting in working life. Neglecting to address age as an issue in the organization could possibly indicate some form of age discrimination: age is getting “the silent treatment”. Silent age discrimination is difficult since it could harbour a variety of attitudes behind the silence: age as a non-topic, age as a taboo, age as something negative, insecurities about dealing with age issues, but also respect for individual integrity and treating everybody in the same way. Skučienė and Moskvina [9] showed, that positive attitudes are not necessarily connected to practice for prolonging working life, even if managers’ attitudes were found by Furunes et al. [21], Kunze et al. [22] and Oude et al. [23] to help in understanding how access to human and financial resources were provided within the organization. In line with previous research, this study finds that although many informants talk about the benefits of older workers, they do not have practices to support the development of older workers. This means that the ambition of age management put forward by Ilmarinen to consider the different strengths of a diverse workforce [4], is still not on the agenda for the organizations in this study.

## 6 Conclusions

This study shows that among the interviewed managers, there is an ambivalence in addressing the issue of age and older workers. In the interviews, the informants prefer to talk about competence and experience rather than age. Singling out employees solely based on age as a variable could create an even stronger stigma around age. Still, it seems the informants in this study did not have elaborated and explicit strategies for competence transfer or development of older workers. The government’s bill to raise the retirement age has not entailed specific activities, measures, or steps. The knowledge gap in their organizations includes laws and regulations around retirement, work capacity assessments, healthy workplaces, aging as a phenomenon and the challenges of older people. By highlighting the state of knowledge and what readiness there is to retain older workers in the investigated organizations, our study shows what needs to be improved for the government’s intentions with an extended working life to be achieved. The rise in the retirement age has only added a few years to working life, without transformational effects in the short term. We do, however, believe that in the long term, a working life stretching over a longer time will call for new ways to manage competence, careers, and human resources.

## 7 Strengths and limitations

The strengths and limitations of this study are discussed in relation to the four aspects of trustworthiness in line with Lincoln and Guba [28] under the heading Trustworthiness in the Method section.

## Ethical approval

Not applicable since this study is not about a vulnerable group of persons or handle any sensitive data.

## Informed consent

All participants gave their informed consent, please see Ethical considerations 3.5.

## Conflict of interest

This study does not report any conflicts of interest.

## Supplementary Material

Appendix
