# Supplementary material for: Older workers and extended working life – Managers’ experiences and age management
Source: Work. 2024 Nov 8;79(3):1323–31. doi: 10.3233/WOR-230468 (PMC11613071; doi:10.3233/WOR-230468)
Supplement: Appendix [file wor-79-wor230468-s001.docx]

Appendix 1

## Interview guide

**Introductory questions**

Tell me little about yourself and your role in the organization.

**Theme 1. Older workers in the organization**

How is the age distribution in you organization? Who is considered old in your organization?

**Theme 2. Retirement**

At what age do most of your workers retire? Usual retirement age 65 or later, to 67?

**Theme 3. Competence transfer and competence development**

Is competence loss due to retiring workers a problem in your organization? Are you working actively with the question and if so, how? If not, why?

**Theme 4. The Government’s proposition about increased retirement age**

Have you heard about the Government’s proposition about increased retirement age? (Tell if they do not know). How do you feel about it? Have you discussed it in your organization?

**Theme 5. Need for knowledge**

Are there any aspects regarding older workers that your organization need more knowledge about?

**Rounding off**

Thank you for your time. Is there something you would like to add?
